# SUCCESSFUL AGING AND FACTORS ASSOCIATED WITH IT AMONG OLD PEOPLE LIVING IN OLD AGE HOMES IN NEPAL

**DOI:** 10.1093/geroni/igae098.1783

**Published:** 2024-12-31

**Authors:** Hom Nath Chalise, Satya Singh

**Affiliations:** Tribhuvan University, Kirtipur, Bagmati, Nepal; Torrens University, Adelaide, South Australia, Adelaide

## Abstract

This study aimed to assess Successful Aging (SA) and its association with Nepali older people. A cross-sectional analytical study was conducted among 206 elderly people of seven old age homes in the Kathmandu district. The interview technique using a structured questionnaire was taken after informed consent. The data collection tool was based on the determinants of SA and the Successful Aging Inventory (SAI) tool. The Geriatric Depression Scale (GDS) was used as an indicator of success in mental health. The ethical review board of Nobel College in Nepal permitted this study. The majority of the respondents (63.5%) were successful agers according to SAI Indicators and represented the SA components such as 90.4% in SP, 87.5% in PC, 93.3% of respondents self-rated health (SRH), 55.8% were involved in mind diversional activities(MDA), 99% have resilience with illness and almost two third of the respondents were mentally healthy according to GDS analysis. Most of the SA was from the 71+ age group (60.6%)with the odds of having success in the 71+ age group being 2.45 times more compared to the 60-70 years age group. Mann Whitney-U test for a non-normally distributed population showed a statistically significant difference found between the Female and Male group for SA, the difference was also found in PC and MDA among SA and Usual Aging. Although the majority of the respondents were successful agers, there needs to be improvement in all aspects especially the mental health of SA components to achieve better SA among older people.

